# STORK: Collaborative Online Monitoring of Pregnancies Complicated with Gestational Diabetes Mellitus

**DOI:** 10.3390/healthcare10040653

**Published:** 2022-03-31

**Authors:** Christos Chatzakis, Dimitris Floros, Anastasios Liberis, Aggeliki Gerede, Konstantinos Dinas, Nikos Pitsianis, Alexandros Sotiriadis

**Affiliations:** 1 Second Department of Obstetrics and Gynecology, Aristotle University of Thessaloniki, 54642 Thessaloniki, Greece; cchatzakis@gmail.com (C.C.); liberis-t@hotmail.com (A.L.); agerede@otenet.gr (A.G.); konstantinosdinas@hotmail.com (K.D.); 2 Department of Electrical and Computer Engineering, Aristotle University of Thessaloniki, 54124 Thessaloniki, Greece; fcdimitr@ece.auth.gr (D.F.); pitsiani@ece.auth.gr (N.P.); 3 Department of Computer Science, Duke University, Durham, NC 27708, USA

**Keywords:** gestational diabetes mellitus, GDM, collaborative online monitoring, mobile healthcare platform, sustainable quality healthcare, data-driven adaptive GDM therapies

## Abstract

Background: A novel digital platform, named STORK, was developed in the COVID-19 pandemic when clinic visits were restricted. A study of its clinical use during the pandemic was conducted. The study aims to advance the state of the art in monitoring and care of pregnancies complicated with gestational diabetes mellitus (GDM) via online collaboration between patients and care providers. Methods: This study involved 31 pregnant women diagnosed with GDM and 5 physicians. Statistical comparisons were made in clinic-visit frequency and adverse outcomes between the STORK group and a historical control group of 32 women, compatible in size, demographics, anthropometrics and medical history. Results: The average number of submitted patient measurements per day was 3.6±0.4. The average number of clinic visits was 2.9±0.7 for the STORK group vs. 4.1±1.1 for the control group (p<0.05). The number of neonatal macrosomia cases was 2 for the STORK group vs. 3 for the control group (p>0.05); no other adverse incidents. Conclusions: The patient compliance with the pilot use of STORK was high and the average number of prenatal visits was reduced. The results suggest the general feasibility to reduce the average number of clinic visits and cost, with enhanced monitoring, case-specific adaptation, assessment and care management via timely online collaboration.

## 1. Introduction

Close monitoring of pregnancies complicated with gestational diabetes mellitus (GDM) is critically important to maternal and neonatal health care, risk prevention and cost reduction [1,2,3,4,5,6,7]. GDM is one of the most common medical complications of pregnancy [8,9,10,11,12,13]. It is recognized as a significant risk factor of adverse perinatal outcomes, long-term obesity, and glucose intolerance in offspring. In recent decades, self-monitoring blood glucose (SMBG) has been widely adopted to provide individual glucose information to the patient as well as to the care team, which includes attending physicians, dietitians, physical training consultants and family members [4,14]. Fundamentally, the care process is a feedback loop between monitoring and intervention. Intervention is by dietary therapy and physical exercise, and when necessary, by pharmacological therapy as well [15]. In adaptation to the monitoring feedback, the therapies are carefully planned or prescribed in concert, mostly by the care provider team. The overall objective is to maintain the patient’s blood glucose level within a target range and small variation.

In conventional practice, patients report their daily SMBG measurements to their attending physicians only at the pre-scheduled walk-in clinic visits, with many days in between. The patient measurements are presented in various forms or formats, manually made or software generated. There is a need of systematic means to register multiple measurements together. We may put the measurements into two categories. The measurements in one category indicate the glycemic status or response variables, such as glucose level, blood pressure, weight change and sleep duration [16]. The measurements in the other category are related to glycemic control variables with dietary therapy, physical exercise and pharmacological therapy, such as the time and amount of carbohydrate intake, the energy expenditure of physical activity, and the time and dose of medicine taken [10].

Self-monitoring and self-recording of daily measurements can be improved and made more convenient with the use of modern digital technologies. Particularly, there is growing attention, analysis and discussion regarding the use of smartphones in the hope to improve patient compliance [17,18,19,20,21,22]. Two other aspects of the care process call for equal or more attention and action. First, the care providers need to be timely informed of the monitoring feedback, in order to make timely assessments of progress or setback and adaptation in intervention therapies. Conventional time lags in obtaining the monitoring feedback should and can be reduced or eliminated [5,6,23]. Second, and more importantly, the daily execution of multiple intricate therapy plans falls primarily on the patient herself, which entails greater effort than daily self-monitoring. The need for the patient to get timely counseling and reinforcement in both daily monitoring and therapy implementation should not be underestimated.

**Figure 1:**
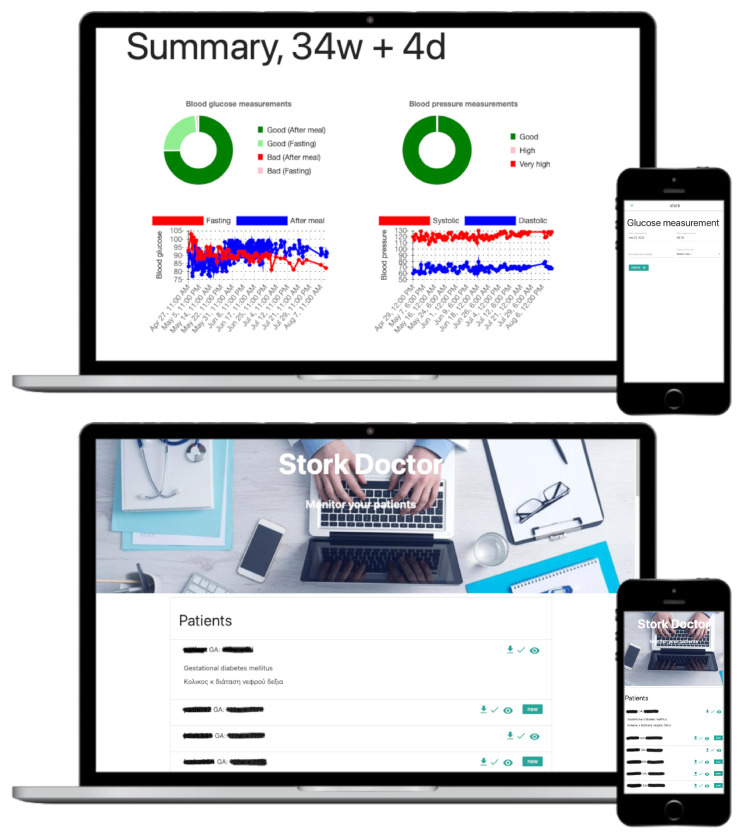
Demonstration of STORK user interface on personal computers and mobile devices. (**Top**): Patient view. An automated summary of feedback in multiple charts and plots. The upper twopie charts show the rates of out-of-target-range measurements of glucose level and blood pressure. The lower two plots display dynamic variations over time in glucose level and blood pressure. (**Bottom**): Doctor view. A scrollable list for selection and retrieval of patient information. Each demo image has an insert of the mobile version on a smartphone at the bottom-right corner. See detailed STORK description in Section 2.1.

**Figure 2:**
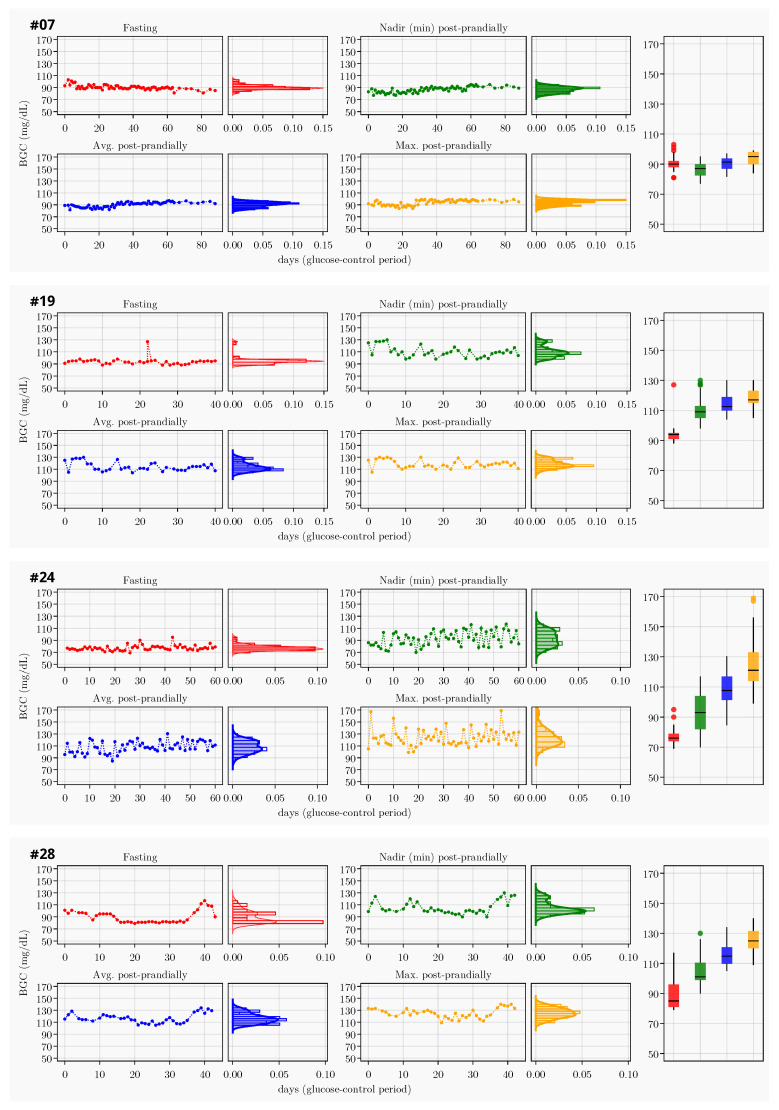
Blood glucose concentration (BGC) profiles of four patients with IDs 07, 19, 24 and 28. The profile for each patient is shown in four time sequences in red, green, blue and yellow (RGBY), their respective histograms and a box-and-whisker chart. The red time sequence and histogram are associated with daily fasting BGC measurements; the green, the daily nadir (minimum) of postprandial measurements; the blue, the daily mean of postprandial measurements; and the yellow, the daily maximum of postprandial measurements. Each histogram is also overlaid with an approximate continuous distribution curve. The box-and-whisker chart summarizes the mean locations and dispersions of the four time sequences. See more comments in Section 3.3 and Section 4.3.

**Figure 3:**
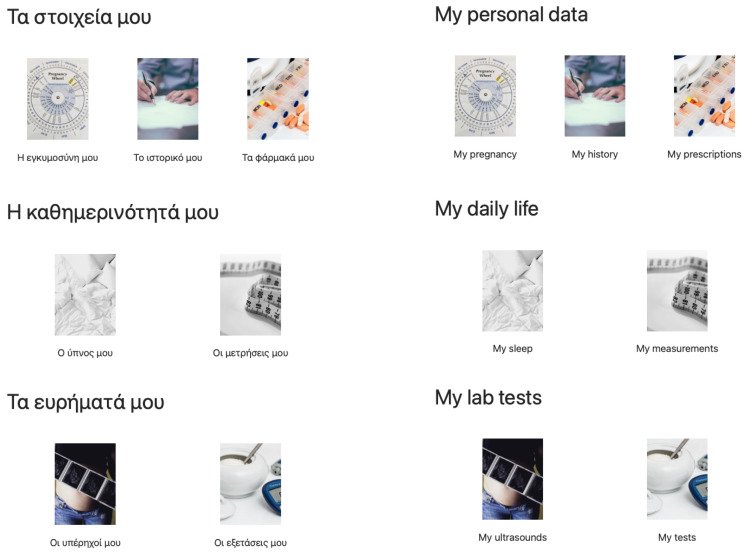
The use of STORK in clinical practice is the first in Greece with online collaborative GDM monitoring. The STORK user interface is bilingual, in Greek and English. STORK has ready functionality for accommodating more languages. See more description of STORK in Section 2.1.

**Figure 4:**
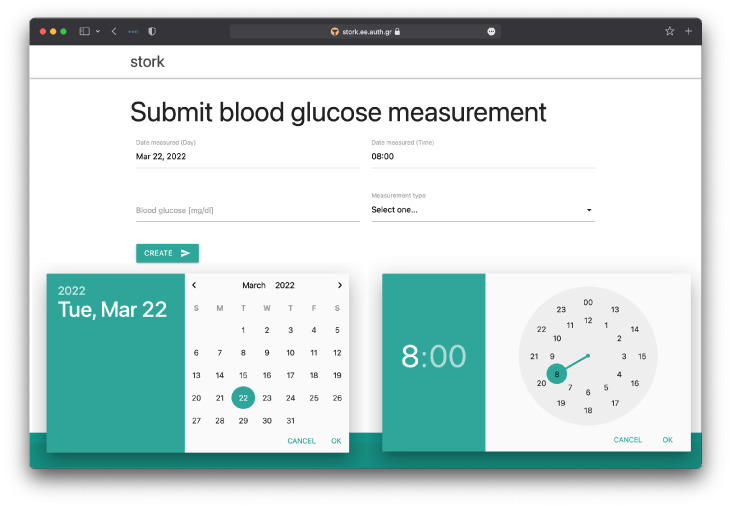
The ease of use is an important criterion for STORK design. The panel for patient submitting blood glucose measurements, for example, hasa pop-up calendar anda clock dial to ease the registration of timestamps, which the usersappreciated. Further ease of use is expected with the advance in automatic data transport between smart devices, see more comments in Section 4.

We advocate closer and constructive collaboration, through every patient-specific care process, between the patient and the care-provider team, above and beyond patient compliance. For technical support of the collaborative compliance principle, we developed a novel digital platform, named STORK, for collaborative online monitoring and counseling between the patient and her care team, see Figure 1, Figure 2, Figure 3 and Figure 4. Among other benefits, STORK re-moves the conventional time lags in reporting, monitoring reviews and counseling, enables co-registration of multiple daily measurements, and encourages and enhances patient compliance.

We report our study on the pilot use of STORK. The study is the first clinical study in Greece on online GDM monitoring with modern digital technologies and with patient-physician collaboration, to our knowledge. The STORK platform is bilingual, in Greek and English, see Figure 3, with ready functionality to accommodate more languages. The pilot study was initiated with the urgent need to sustain and continue monitoring and care of GDM-complicated pregnancies during the COVID-19 pandemic peak period when walk-in clinic visits were restricted or outweighed by the increased likelihood of exposure to the virus [24,25,26,27,28,29,30,31]. The broader goal of the study is to advance the state of the art in GDM monitoring and intervention. This goal includes sustainable quality healthcare in extraordinary times or challenging circumstances as well as healthcare in ordinary times, in urban and rural areas.

In the rest of the paper, we describe STORK functionalities, the pilot use of STORK, and statistical analysis with comparison to a historical control group. We discuss on the digital technologies necessary for supporting the distinctive properties of STORK, desirable and plausible extensions, and the need to expand the study on the efficacy of using STORK-like collaboration platforms.

## 2. Materials and Methods

### 2.1. STORK Platform

We designed and developed a novel digital platform, named STORK (https://stork.ee.auth.gr, online since January 2020, accessed on 25 March 2022), to enable collaborative online monitoring and timely adaptive care of pregnancies complicated with GDM. The platform is easily accessible by a patient and her attending physician(s) from anywhere at any time, via an internet connection, by computers or mobile digital devices such as smartphones and tablets. Patient privacy is ensured with advanced computer security technologies.

We describe the key functionalities of STORK from the patient perspective as well as the physician perspective. The user interface guides each patient to submit, with great ease, multiple measurements of glycemic status variables and glycemic control variables as described in Section 1. The ease of use is an important criterion in STORK design before automatic data transport between smart sensor devices and data analytics devices becomes economically and widely available. For example, STORK makes it easy to register timestamps with a quick click in a pop-up calendar and a simple dial of the clock hand, see Figure 4. In return, the patient can get timely feedback, in text and multiple charts, based on the recent glucose profile, progress or setback in glucose level control. Each chart provides a view of the response of a particular glycemic status variable (e.g., the glucose level) to the change in a particular glycemic control variable (e.g., carbohydrate intake). See Figure 1. There are two types of feedback. One is generated by the digital expert recommendation system within STORK. The other is timely advice offered by the attending physician when the need arises, without delay until the next scheduled clinic visit.

The doctor’s attention is raised either by a direct request from the patient or by the expert and data-driven recommendation system within STORK. For example, a message is sent to the physician when the glucose or blood pressure measurements of a patient are off target, or when the measurements are not submitted for more than two days. There are also patient-specific conditions detailed by the physician/expert for STORK to track and report. Furthermore, a physician can retrieve, with ease, from the platform a coherent glucose profile of a patient for a comprehensive patient-specific review, see Figure 1 and Figure 2. In comparison, the conventional process limits the physician review in time, location and contents. The review time and location are constrained by brief time windows at pre-scheduled clinic visits, many days in between. The review contents are limited to the snapshots or summaries reported by patients. STORK also provides the convenience for physicians to integrate patient submitted data with lab data such as ultrasound examination results. Empowered by the STORK analytics engines, physicians can make more effective and efficient use of their expert knowledge, without being burdened or frustrated by incomplete, incoherent or increased data.

The STORK functionalities are supported by several subsystems for access control, data management, data processing, analytic engine and automated recommendation.

### 2.2. Pilot Use of STORK

The pilot clinical use of STORK was at the Hippokration General Hospital (HGH), the Second Department of Obstetrics and Gynecology, Aristotle University of Thessaloniki, in Greece, during the COVID-19 pandemic. It was approved by the Scientific and Ethical Board at HGH (Reference No. E.Σ. 323/24620), in accordance with the Declaration of Helsinki. Specifically, the pilot study period was from February to September 2020.

The pilot study participants were women diagnosed with GDM. The GDM diagnosis followed the recommendation by the International Association of Diabetes and Pregnancy Study Groups (IADPSG) [32]. Specifically, a 75 g two-hour oral glucose tolerance test (OGTT) was carried out at 24–28 weeks of gestation. Pregnant women with blood glucose values equal to or greater than 92 mg/dL at fasting, 180 mg/dL at 60 min and 153 mg/dL at 120 min after consumption of 75 g of glucose were diagnosed with GDM. The patient submits four blood glucose measurements per day, one fastingmeasurement in the morning and threemeasurements postprandially—from one to two hours after each meal. The study excluded pregnancies complicated by pre-existing diabetes mellitus (type 1 or 2), hypertensive disorders of pregnancy, chromosomally abnormal fetuses with or without structural defects and fetal growth restriction.

### 2.3. Historical Control Group

All the pregnant women with GDM at HGH in the study period were recommended to use STORK. It was implausible and against the do-no-harm principle to set up a randomized control group without using STORK in the COVID-19 pandemic. For comparative analysis, we made use of a historical control group by the archived patient records at HGH. The group consisted of pregnant women diagnosed with GDM from February to September in 2019, the same period on the annual calendar as the study period but a year earlier. The historical control group excluded pregnancies complicated by pre-existing diabetes mellitus (type 1 or 2), hypertensive disorders of pregnancy, chromosomally abnormal fetuses with or without structural defects and fetal growth restriction. In the rest of the paper, we refer to the group of patients participating in the pilot study as the STORK group and the historical control group as the control group. All quantitative information reported in this paper about the control group was retrospectively searched and extracted from the HGH records.

### 2.4. Variables

We report three primary outcome indicator quantities, along with detailed pregnancy-delivery conditions and neonatal outcomes, and uniquely with patient-specific blood glucose concentration statistics in addition. The first is the average number of daily measurements submitted by the STORK participants. This number indicates how well STORK was functioning, appreciated and utilized. The second indicator is the average number of walk-in clinic visits. Reducing the number of walk-in clinic visits and the cost is a long-term benefit and impact of STORK, not only for the urgent need during the COVID-19 pandemic. The third quantity reflects the outcome of risk prevention through monitoring, counseling and care management. A critical indicator is the number of adverse incidents in eachcategory of intrauterine death, perinatal death, neonatal macrosomia (birth weight greater than 4000 g or higher than the 90th percentile), shoulder dystocia and neonatal hypoglycemia.

### 2.5. Statistical Analysis

Categorical variables were summarized by respective percentages. Non-categorical variables with normal distributions were summarized by the mean values and the standard deviations; with non-normal distributions, by the median values and the interquartile ranges. The t-test or Mann–Whitney U test was used to compare non-categorical variables; the chi-square or Fisher’s exact test was used for pairwise comparisons of categorical variables.

## 3. Results

### 3.1. Participants

The study involved 31 pregnant women diagnosed with GDM and 5 physicians. There was a total of 33 pregnant women diagnosed with GDM during the pilot study period. They were all recommended to participate in the pilot use of STORK. Only 2 of them declined, claiming their lack of experience with computers or mobile devices. All 31 participants gave their informed consent. The control group has 32 patients.

### 3.2. Descriptive Data

Statistically descriptive information of the STORK group and the control group is shown in Table 1. In particular, the average gestational age was 28.1±1.4 for the STORK group vs. 27.5±2.2 for the control group (p>0.05). The two groups were compatible in size, demographics, anthropometrics and medical history. There was no significant difference between the two groups in any variable in Table 1.

### 3.3. Main Results

The average number of submitted patient measurements per day was 3.6±0.4. The average calculation takes into account that the time duration of each STORK study participant is inevitably specific to the particular patient. The average number of walk-in clinic visits was 2.9±0.7 for the STORK group vs. 4.1±1.1 for the historical control group (p<0.05).

There was no statistically significant difference between the STORK group and the control group in glycemic status variables, pregnancy and delivery conditions, and neonatal outcomes; see Table 2 and Table 3. No participant in the STORK group tested positive for COVID-19. There were 2 cases of neonatal macrosomia among the STORK group; there were 3 cases of neonatal macrosomia among the historical control group (p>0.05). There was no other adverse incident among the STORK group, nor among the control group.

More compelling results of the pilot study are illustrated in Figure 2, which depicts the blood glucose concentration (BGC) profiles of four participants. The profile for each patient consists of four time sequences in red, green, blue and yellow (RGBY), their respective histograms and a box-and-whisker chart. The red time sequence and histogram are associated with the daily fasting measurements; the green, the daily nadir (minimum) of postprandial measurements; the blue, the daily mean of postprandial measurements; and the yellow, the daily maximum of postprandial measurements. Each histogram is also approximated by a continuous distribution curve. The box-and-whisker chart summarizes the mean locations and dispersions of the four time sequences. The BGC profile of patient No. 7 shows the least variation in both fasting and postprandial measurements and the smallest gap between fasting and postprandial measurements. The BGC profile of patient No. 24 exhibits a large variation in the nadir postprandial measurements and a wide range in daily measurements.

## 4. Discussion

### 4.1. Key Results

The patient compliance with the pilot use of STORK was high and the average number of prenatal visits was reduced. The results suggest the general feasibility to reduce theaverage number of clinic visits and cost, with enhanced monitoring, case-specific adaptation, assessment and care management via timely online collaboration.

The most remarkable and unique results are the data and inferred information made available via STORK to both patients and physicians, as shown in Figure 2. These results are unprecedented in clinical GDM monitoring and management. The measurement data, at daily frequency, with dynamic trajectories and accumulated volume, were previously unavailable to the attending physicians. Even in previously reported studies of remote GDM monitoring with smartphones [17,18,19,20,21,22], patients were expected to make unilateral efforts in daily measurement, assessment and management. STORK has changed that status quo.

### 4.2. Limitations

The main limitation of the pilot study was the lack ofa randomized setting. It was implausible and against the do-no-harm principle to set up a randomized control group without using STORK during the COVID-19 pandemic. We expect the study to be expanded in several aspects, such as in larger size, diverse demographics, anthropometrics, medical history, also in different environments, seasons and regions.

### 4.3. Interpretation

About the primary outcomes. Every participating patient succeeded, during her time period of using STORK, in submitting her daily measurements, as prompted and guided by the STORK user interface, see the patient view in Figure 1. The patient’s active participation is in part due to the STORK guidance and ease of use, and in part due to the pandemic circumstance. The high compliance is satisfactory, thanks to the patient-doctor collaboration via STORK. Any reduction in the average number of clinic visits shall be the result of quality care, not the driving objective. The attending physicians can easily and timely locate and track pregnancies with measurements out of the target range, or unusual variation and trajectory. They can offer timely advice via the STORK message system, out of clinic visit hours. See the doctor view in Figure 1.

About the broader impacts of the pilot study. (i) A modest reduction in the average number of clinic visits implies a substantial reduction in healthcare cost and stress on multiple parties involved. (ii) The study was initiated and carried out during the COVID-19 pandemic, clinic visits were severely restricted, or the benefits of clinic visits were outweighed by the higher likelihood of exposure to the virus. The use of STORK enabled continuous monitoring and care in the extraordinary, challenging time. However, the study is not limited to such extraordinary times. STORK can be used for general healthcare in ordinary times, in urban and rural areas, given the far-reaching presence and prevalence of modern digital communication technologies [7,16,33,34]. (iii) The concept and platform of STORK signal a shift to advanced GDM monitoring and management with bilateral information exchange, closer monitoring and analytic feedback, informed assessment and understanding, and reinforced collaboration.

Additional remarks on the study setting and comparison results. (1) The compatibility between the STORK group and the control group, as described in Section 2.3, is fortunate and important to the statistical comparisons and results, considering that both group sizes are small. The two groups are determined by the actual patient populations at the clinic in 2019 and 2020, respectively, not by design or random selection. (2) Ideally, we wouldalso like to make statistical comparisons on the measurements, such as the mean and variation of blood glucose level, the mean and variation of blood pressure, the rate of out-of-target-range measurements, carbohydrate intake patterns, physical activity and sleep patterns. A major obstacle lies in the discrepancy in temporal data resolution between the STORK group and the control group. There are two feasible ways to make comparisons at the same temporal resolution level. At the finer level, we need the daily measurements for the control group, which were unavailable from the past records. At the coarser level, ideally, we wish to have comparable intervals in clinic visits between the two groups. Due to the pandemic situation, the clinic visits for the STORK group were irregular and farther apart in between.

## 5. Conclusions

The STORK platform is distinct in its design principle and functionalities. Other existing studies on the use of smartphone technologies center mostly on one-way evaluation of patient compliance [17,18,19,20,21,22]. With STORK, we advocate active and constructive online collaboration, above and beyond patient compliance. STORK is thereby designed to support two-way communication and data transactions as described in Section 2.1. We undertook more demanding tasks in STORK development. Among other technical challenges, the top three are data integrity, privacy (and confidentiality), and security (DIPS). We ensure DIPS in STORK with advanced digital technologies—data encryption, access authentication and authorization, and data transaction blockchain.

There are multiple aspects in further STORK study. On the side of digital healthcare technologies, the STORK functionalities can be upgraded and extended in several ways for more convenient and effective use. In particular, measuring and recording devices constantly evolve [4,35]. The current patient-device interface can be upgraded to accommodate automatic data transfer between smart sensor devices and data analytic devicesin the foreseeable near future. With the increasing diversity in sensor devices and data formats, STORK can also be equipped with functions for converting data to a unified format.

On the clinical side, we intend to expand the STORK study in size by involving more physicians and GDM patients. With a sufficiently larger number of participants, we will be able to conduct randomized clinical trials. As the GDM treatment, or lack of it, has a long term impact on both the newborn and the mother, we also intend to extend the use of STORK in time to postnatal periods [3,9,12], not necessarily at daily frequency. Such data and analysis will be valuable to longitudinal GDM studies.

Digital healthcare platforms such as STORK have additional benefits to both clinical practice and basic research. The basic research on GDM study can be extended from research laboratories [36,37,38] to clinics and patient homes. One can retrieve and review archived data for a comprehensive analysis with integrated data. When the deployment of such healthcare platforms becomes common and prevalent, no longer limited to small group studies, researchers can make fundamental studies across diverse patient groups and diverse environments.

## Figures and Tables

**Table 1:** Basic statistic information of the stork group (Section 2.2) and the historical control group (Section 2.3). ART stands for assisted reproduction techniques. SD stands for standard deviation.

	STORK Group	Control Group
**Mean (SD)**	**31 in Total**	**32 in Total**
Maternal age (years)	31.4 (±5.3)	32.1 (±4.8)
Gestational age (weeks)	28.1 (±1.4)	27.5 (±2.2)
BMI before pregnancy	25.4 (±6.5)	24.8 (±6.1)
BMI at delivery	30.7 (±5.7)	31.2 (±5.3)
Weight gain (kg)	14.5 (±8.1)	15.8 (±7.4)
**Total Number (Percentage)**		
Smoking	3 (9.7%)	5 (15.6%)
ART	2 (6.5%)	3 (9.4%)
Parity I	16 (51.6%)	14 (43.8%)
Parity II	10 (32.2%)	10 (31.2%)
Parity III	3 (9.7%)	5 (15.6%)
Parity IV	2 (6.5%)	3 (9.4%)
GDM in previous pregnancy	5 (16.1%)	6 (18.8%)

**Table 2:** Glycemic status.

	STORK Group	Control Group
**Non-Categorical Variables, Mean and Range (SD)**	**31 in Total**	**32 in Total**
OGTT fasting value, mg/dL	93.8 (±10.8)	91.7 (±14.7)
OGTT 60-min value, mg/dL	186.1 (±31.8)	178.0 (±38.3)
OGTT 120-min value, mg/dL	129.3 (±25.9)	142.1 (±32.5)
**Categorical Variables, Total Number and Percentage (%)**		
Insulin treatment	5 (16.1%)	5 (15.6%)

**Table 3:** Pregnancy and delivery conditions and neonatal outcomes.

	STORK Group	Control Group
**Non-Categorical Variables, Mean and Range (SD)**	**31 in Total**	**32 in Total**
Gestational age at delivery, (weeks)	38.5 (±1.3)	38.1 (±1.5)
Birthweight (g)	3298 (±568)	3167 (±495)
**Categorical Variables, Total Number and Percentage (%)**		
Antenatal corticosteroids	0	0
Caesarean delivery	12 (38.7%)	14 (43.8%)
COVID-19 positive	0	N/A
Episiotomy	7 (22.6%)	5 (15.6%)
Emergent cesarean delivery	0	0
Gestational hypertension	0	1 (3.1%)
Hypoglycemia of newborn	0	0
Induction of labor	0	0
Instrumental delivery	0	0
Neonatal death	0	0
Neonatal macrosomia	2 (6.5%)	3 (9.4%)
NICU admission	0	0
Normal vaginal delivery	0	0
Phototherapy	0	0
Polyhydramnios	1 (3.2%)	1 (3.1%)
Preeclampsia	0	0
Respiratory morbidity	0	0
Shoulder dystocia	0	0
Third-/fourth-degree perineal tear	0	0

## Data Availability

Not publically available.

## Abbreviations

The following abbreviations are used in this manuscript:

ART	assisted reproduction techniques
BGC	blood glucose concentration
DIPS	data integrity, privacy and security
GDM	gestational diabetes mellitus
HGH	Hippokrateion General Hospital
IADPSG	International Association of Diabetes and Pregnancy Study Groups
OGTT	oral glucose tolerance test
SD	standard deviation
SMBG	self-monitoring blood glucose
